# Enhancing the cultivation of *Salicornia fruticosa* with agroindustrial compost leachates in a cascade cropping system: evaluating the impact of melatonin application

**DOI:** 10.3389/fpls.2024.1441884

**Published:** 2024-09-09

**Authors:** Almudena Giménez, Victor M. Gallegos-Cedillo, Rachida Rania Benaissa, Catalina Egea-Gilabert, Angelo Signore, Jesús Ochoa, Nazim S. Gruda, Marino B. Arnao, Juan A. Fernández

**Affiliations:** ^1^ Department of Agronomical Engineering, Technical University of Cartagena, Cartagena, Spain; ^2^ Department of Soil, Plants and Food Sciences, University of Bari Aldo Moro, Bari, Italy; ^3^ Department of Horticultural Sciences, Institute of Crop Science and Resource Conservation, University of Bonn, Bonn, Germany; ^4^ Phytohormones and Plant Development Lab, Department of Plant Biology (Plant Physiology), Faculty of Biology, University of Murcia, Murcia, Spain

**Keywords:** biostimulants, primary crop, secondary crop, halophytes, melatonin, leachates, nitrogen use efficiency, water use efficiency

## Abstract

Cascade cropping systems (CCS) utilize leachate from a primary crop to grow secondary crops and enhance the efficient use of water and fertilizers in areas with scarce water resources. A preliminary study investigated the effect of melatonin in a cascade cropping system to potentially improve plant tolerance to abiotic stresses. This study aimed to cultivate *Salicornia fruticosa* in this cropping system to reduce nutrient discharge and assess the impact of exogenous melatonin on Salicornia growth and quality. The CCS included a primary crop of Salicornia grown in an agro-industrial compost or peat. Leachates from these media were used to cultivate the same plant once again in a floating system under four treatments: compost leachate (T1), peat leachate (T2), 100% nutrient solution (NS) (T3), 50% NS (T4) strength. Four concentrations of exogenous melatonin were applied in foliar spray: 0, 100, 200, and 400 µM. Melatonin application increased yield, with the highest values observed when plants were grown in T1. Water use efficiency was also maximized in T1 and with both 200 and 400 µM melatonin applications. The highest nitrogen use efficiency was achieved in plants grown in peat leachate. The lipid membrane damage was assessed revealing that plants grown in compost leachate exhibited the lowest MDA values regardless of melatonin concentrations. The accumulation of some antinutritional compounds (nitrate, oxalate, and sodium) were the highest in those plants grown in compost leachate. Overall, shoots grown in peat leachate exhibited the best phytochemical profile (total phenol content, total flavonoids, and antioxidant capacity), with peak values in plants treated with 200 µM melatonin. These findings suggest that *S. fruticosa* can be effectively cultivated using leachate from a previous crop in a floating system and that exogenous melatonin application enhances the yield and nutritional quality of Salicornia shoots.

## 1 Introduction

The intensification of agricultural practices to meet global food demands has resulted in unintended environmental consequences. One pressing concern is the elevated levels of plant nutrients in water bodies due to agricultural runoff. This contamination disrupts aquatic ecosystems and poses potential health risks.

Nutrient recovery from agricultural drainage emerges as a promising solution for sustainable wastewater management. This approach can mitigate environmental pollution by capturing valuable nutrients before contaminating water bodies. It also enables a more circular economy, promoting resource efficiency and social well-being (Ye et al., 2020). As a result, the focus on water and nutrient recovery has intensified, facilitating nutrient recycling and reuse, minimizing fertilizer consumption, and yielding positive environmental outcomes in greenhouse horticulture (Thompson et al., 2020; Neocleous and Savvas, 2022).

Closed soilless cultivation systems and, mainly, cascade cropping systems (CCS), i.e., systems that collect the drainage from the primary crop to cultivate secondary crops, are of particular interest for sustainable horticulture in naturally dry, water-scarce, and nutrient-poor environments like those along the coast of Mediterranean countries (Santos et al., 2022). The primary emphasis on CCS is on its integration with the fertigation system and its ability to enhance the efficient use of water and fertilizers (Mielcarek et al., 2024). Implementing closed-loop nutrient cycles in soilless culture systems has significantly increased circularity and resource efficiency. This, in turn, leads to significant ecosystem benefits and helps mitigate the impact of climate change (Bisbis et al., 2019; Rufí-Salís et al., 2020a).

Recently, several studies have been conducted in the Mediterranean region using this pattern of production systems (Elvanidi et al., 2020; Faliagka et al., 2021; García-Caparrós et al., 2021; Karatsivou et al., 2023). In these studies, the performance of plants receiving drainage water has been compared to that of plants receiving fresh nutrient solutions (Santos et al., 2022; Gómez et al., 2023).

Overall, the results of the above experiments indicate that, although yield reductions may occur, such drawbacks are offset by increased efficiency in the use of water and nutrients provided by these systems, consistently reducing their environmental impact, water footprint, and the emission of polluting ions such as nitrates and phosphates, compounds especially hazardous in vulnerable zones as the Mediterranean basin (García-Caparrós et al., 2018; Massa et al., 2010).

On the other hand, growing media can affect yield in CCS. However, finding alternatives to peat harvesting is essential, as it significantly impacts ecosystems and contributes to carbon emissions that cause the greenhouse effect (Gruda et al., 2019; Gruda, 2022; Gruda et al., 2024a).

Compost from agrifood industry waste is a viable alternative to peat in sustainable soilless production (Giménez et al., 2019). This replacement contributes to resource recovery, a key principle of the circular economy (Razza et al., 2018). Furthermore, adding directly brewed compost extract to the nutrient solution has improved crop yield and vegetable quality (Giménez et al., 2020). Sea fennel plants showed a higher nutritional quality when they were grown using the leachates derived from the wild rocket growing medium (Gómez et al., 2023). Therefore, leachates from a crop grown in compost as a growing medium could improve crop performance in a cascade crop system (Amoruso et al., 2023).

Studies show that CCS efficiency hinges on two key features (Katsoulas and Stanghellini, 2019; Kumar and Cho, 2014). First, strategically chosen crops: a high-nutrient upper tier paired with a lower tier that thrives on the recycled solution (Elvanidi et al., 2020). Second, salt tolerance in the lower tier to handle accumulated salts (Rufí-Salís et al., 2020b; Gruda et al., 2024b).

Controlled environments and soilless culture enable precise adjustments of nutrient profiles in vegetables through the regulated application of moderate salinity. This metabolic reprogramming allows vegetables to enhance their production by synthesizing phytonutrients and other health-promoting compounds (Gruda et al., 2024b). Moderate salinity is widely used to enhance various vegetables’ quality and nutritional properties. This practice regulates salt levels or nutrient solution concentrations to induce physiological and molecular responses that improve nutritional quality and promote the accumulation of beneficial bioactive compounds in protected cultivation (Gruda, 2009; Gruda et al., 2018, **2024b**).

Glasswort (*Salicornia* spp.), a salt-resistant species, could be an excellent candidate for inclusion in saline drainage water reuse systems (Chaturvedi et al., 2021). This species has been recently introduced into the European market as a vegetable with a high nutritional value as a source of proteins, vitamins, carotenes, and phenols with potential antioxidant activity (Antunes et al., 2021). Furthermore, Salicornia plants irrigated with high concentrations of seawater increased their content of antioxidant compounds (polyphenols, β-carotene) and proteins, thus increasing their nutritional value (Ventura et al., 2011).

Melatonin (*N*-acetyl-5-methoxytryptamine) is an indole molecule discovered in cows and humans in 1958 and 1959 and in plants in 1995; also described in all living organisms, from prokaryotes to humans as an endogenous metabolite with relevant functions. In plants, melatonin is involved in several physiological actions such as germination, rooting, growth, photosynthesis, water and CO_2_ uptake, flowering, fruiting, parthenocarpy, ripening, and senescence (Arnao and Hernández-Ruiz, 2020). As a relevant role, it was highlighted as an antioxidant regulating stress responses (Iqbal and Khan, 2022). Melatonin is an excellent enhancer of tolerance (resilience) of plants to abiotic stressors such as drought, flooding, salinity, cold/heat, toxic agents, heavy metals, UV radiation, etc (Arnao and Hernández-Ruiz, 2014; Moustafa-Farag et al., 2020; Altaf et al., 2021; Arnao and Hernández-Ruiz, 2022a). The use of melatonin in crops has the potential to increase food production, improve its postharvest quality, reduce water loss, and increase color and secondary metabolites such as vitamins, carotenoids, glucosinolates, phenolics such as flavonoids, among others, through the modulation of primary and secondary metabolisms (Arnao et al., 2022b). It also improves endogenous pathogen response against infections by bacteria, fungi, and viruses (Tiwari et al., 2021; Zhao et al., 2021; Hernández-Ruiz et al., 2023; Giraldo-Acosta et al., 2022).

Hence, in this study, we aim to contribute to a more circular and resource-efficient agricultural approach by investigating a closed-loop CCS. Specifically, the objectives were to (i) evaluate the performance of *Salicornia* grown in a secondary floating system by using leachate deriving from two distinct growing media of a *Salicornia* primary crop and (ii) investigate the impact of melatonin as a biostimulator of plant growth and stress control, in such conditions.

## 2 Material and methods

The experiments were conducted at the Experimental Agro-Food Station of the Technical University of Cartagena (lat. 37°41’ N; long. 0°57’ W) in an unheated greenhouse covered with thermal polyethylene for both primary and secondary crops.

### 2.1 Plant material and growing conditions for the primary crop of *Salicornia fruticosa*

*Salicornia fruticosa* seeds obtained from Viveros Muzalé, Fortuna (Murcia) were sown on 19 November 2022, in Semirec^®^ plastic trays measuring 63.2 x 31.5 cm (220 cells, cylindric cell volume 12 cm^3^). They were filled with commercial peat-based substrate (Peat moss-Turbas y Coco Mar Menor, S.L., Spain). The seeds were kept in a germination chamber at 25°C for ten days before being transferred to a growth chamber, where they were cultivated for two months. The growth chamber maintained a temperature of 23°C, a relative humidity of 65%, and a photoperiod of 12/12h. The photosynthetic photon flux density (PPFD) resulted in a daily light integral (DLI) of 7 mol day^-1^.

When the plants reached a height of 5 cm, they were transplanted into metallic gutters with a pyramidal trunk section filled with peat or an agro-industrial compost as a growing media. Their physical and physicochemical characteristics have been described in previous reports (Signore et al., 2023; Gómez et al., 2023). Plant density was 49 plants m^-2^. After transplanting, the following nutrient solution (NS) (Signore et al., 2023) was applied daily to the plants. The concentration of the NS during the crop cycle was half strength during the first week (19 to 26 March 2023) and full strength from the second week to the end of the experiment (24 May 2023). The electrical conductivity (EC) was 1.47 dS m^-1^ during the first week and 2.37 dS m^-1^ for the rest of the cycle. An automated system was utilized to establish a similar moisture level in the growing media during the experiment. The leachates of compost and peat were collected in separate tanks, disinfected using an ultraviolet system, and analyzed at the end of the growing cycle (**Table 1**).

**Table 1:** Initial chemical composition (mM) of the nutrient solution used.

Ion	T1	T2	T3	T4
**NO_3_^-^**	6.89 ± 0.06 a	6.27 ± 0.01 b	4.85 ± 0.02 c	2.7 ± 0.00 d
**PO_4_^3-^**	0.13 ± 0.01 c	1.63 ± 0.00 a	1.65 ± 0.01 a	0.83 ± 0.00 b
**SO_4_^2-^**	9.15 ± 0.33 a	7.35 ± 0.04 b	2.28 ± 0.01 c	1.36 ± 0.00 d
**Cl^-^**	6.32 ± 0.05 a	5.39 ± 0.00 b	3.11 ± 0.02 c	3.15 ± 0.01 c
**NH_4_^+^**	2.11 ± 0.02 b	1.16 ± 0.00 d	3.23 ± 0.02 a	1.57 ± 0.00 c
**K^+^**	12.54 ± 0.33 a	6.03 ± 0.17 b	5.57 ± 0.01 b	2.75 ± 0.01 c
**Ca^2+^**	8.68 ± 0.26 a	6.87 ± 0.09 b	1.8 ± 0.00 c	1.38 ± 0.00 c
**Mg^2+^**	10.6 ± 0.32 a	6.84 ± 0.09 b	1.53 ± 0.00 c	1.09 ± 0.07 c
**Na^+^**	7.67 ± 0.25 a	7.56 ± 0.05 a	3.24 ± 0.04 b	3.24 ± 0.00 b
**pH**	8.24 ± 0.02 a	4.59 ± 0.02 c	6.38 ± 0.06 b	6.44 ± 0.12 b
**EC (dS m^-1^)**	4.54 ± 0.15 a	3.75 ± 0.15 b	2.11 ± 0.03 c	1.36 ± 0.01 d

T1, compost leachate; T2, peat leachate; T3, 100% nutrient solution (NS) and T4, 50% NS strength. All data represent the mean value ± SE (n=3). Different letters in rows indicate significant differences among treatments at p ≤0.05 (Tukey´s test).

### 2.2 Plant material and growing conditions for the secondary crop of *Salicornia fruticosa*

*Salicornia fruticosa* seeds were sown on 11 October 2023 in styrofloat trays measuring 0.6 m × 0.41 m filled with a commercial peat-based substrate (peat moss-Turbas y Cocos Mar Menor S.L., Spain). The trays were kept in a germination chamber at 25°C for nine days and a relative humidity of 90%, in darkness before being transferred to floating beds (1.35 m × 1.25 m × 0.2 m), where trays were floating on fresh tap water with an EC of 1.1 dS m^−1^ and a pH of 7.8. Aeration was ensured using a pump and a pipe trellis system placed on the bottom of each bed. On 10 November 2023, the plants were thinned to the definitive density of 4,000 plants m^-2^. One month after sowing, the tap water of each bed was replaced with 180 liters of the following treatments: compost leachate (T1), peat leachate (T2), 100% nutrient solution (NS) (T3), 50% NS (T4) strength. The NS used to prepare (T3 and T4) was a similar that employed by Signore et al. (2023). Four concentrations of exogenous melatonin were applied 56 days after sowing to plants: 0 µM melatonin (MEL0), 100 µM melatonin (MEL100), 200 µM melatonin (MEL200), or 400 µM melatonin (MEL400). Melatonin was dissolved in 10 mL of ethanol to produce a 1 mM stock solution and then diluted in miliQ water to generate the working concentrations. In the case of MEL0 concentration, only miliQ water was applied. Three melatonin spray applications were carried out on shoots, and there was a separation of 10 days among them.

The initial chemical composition of the NS and leachates were analyzed in terms of EC, pH, and elements such as N (as NO_3_^-^ and NH_4_^+^), PO_4_^3-^, SO_4_^2-^, K^+^, Ca^2+^, Mg^2+^, Na^+^, Cl^-^ by ion chromatography (**Table 1**) using a Metrosep A SUPP 5 column (Metrohm AG, Zofingen, Switzerland) at a flow rate of 0.7 mL min^-1^ for anions and a Metrosep C 2-250 column at a flow rate of 1.0 mL min^-1^ for cations. The ion concentration, pH, and the EC of the NS were analyzed weekly.

### 2.3 Plant parameters

The harvest was conducted on 5 February 2024, when the shoots grew to approximately 10 cm in height to facilitate their later process as a minimally processed product. Several measurements were taken, including fresh (FW), dry weight (DW), yield, dry matter percentage (DM), plant height, root length, succulence (shoot FW/DW), and shoot area (SA).

Shoot area (SA) was determined using the software ImageJ (Schneider et al., 2012), specific shoot area (SSA) (Equation 1), shoot area ratio (SAR) (Equation 2) and shoot/root ratio was calculated as the shoot and root dry weights of a plant (Gallegos-Cedillo et al., 2021).


(1) $$\text{SSA}=\frac{SA\left(cm^{2}\right)}{Shoot\;dry\;mass\;\left(g\right)}$$

(2) $$SAR=\frac{SA\left(cm^{2}\right)}{Total\;dry\;weight\;\left(g\right)}$$

NS consumption was calculated as the difference between the input, the volume supplied to the system, and the output, the volume left in the system at the end of the crop cycle. Water use efficiency (WUE) (kg m^-3^) was calculated as a ratio between yield (kg m^-2^) and NS consumption (m^3^ m^-2^). In addition, the nitrogen use efficiency (NUE) was calculated as the ratio of yield (kg m^-2^) to nitrogen removed from the crop (kg g^-1^). The N concentration in the nutrient solution (NS), together with the volume of NS, was used to calculate the amount of N given to the crop. Then, the concentration of N in the leachate, multiplied by the leachate volume, was used to determine how much N was not absorbed. Finally, the first amount was subtracted to the latter one, to calculate how much N was absorbed by the crop.

### 2.4 Color

A colorimeter (Minolta CR-400 Series, Ramsey, NJ, USA) was used to determine the color of shoots on three spots on the upper side of ten shoots from each duplicate. The CIELab system’s tristimulus parameters (L*, a*, b*) were utilized to compute the hue angle = arctan (b*/a*) and chroma C*= (a*^2^ + b*^2^) ^ ^(1/2)^.

### 2.5 Determination of chlorophyll and carotenoid contents

A methanolic extract was obtained with 150 mg of fresh weight sample in 1.5 mL of MeOH to determine chlorophylls and carotenoids. The equations developed by Lichtenthaler and Buschmann (2001) were used to determine total chlorophyll, and total carotenoid contents were expressed as mg kg^-1^ FW.

### 2.6 Determination of malondialdehyde

Malondialdehyde (MDA) content, a product of lipid peroxidation, was estimated using the thiobarbituric acid (TBA) method, which is used to determine cell membrane damage (Niehaus and Samuelsson, 1968). The expression of the results was milligrams of MDA per gram of fresh weight (mg MDA g^-1^ FW). The determinations were made in triplicates.

### 2.7 Plant ion contents

The inorganic ions were extracted using a 0.2 gr dry shoot sample with 50 mL of distilled water. After the extraction, the samples were agitated in an orbital shaker (Sturat SSL1, Stone, UK) for 45 min at 100 rpm at 50°C. The resulting chemical composition of the Salicornia treatments was analyzed in terms of N (as NO_3_^-^), K^+^, Ca^2+^, Mg^2+^, Na^+^, Cl^-^, and SO_4_^2-^ by ion chromatography, as indicated above.

### 2.8 Total phenolic content, total flavonoid content, and total antioxidant capacity

The total phenolic content (TPC) and the total flavonoid content (TFC) were determined as described by Martínez-Zamora et al. (2021). For TPC, 19 μL of the sample extract was mixed with 29 μL of 1 N Folin-Ciocalteu reagent and 192 μL of 0.4% Na_2_CO_3_ and 2% NaOH, measuring the absorbance at 750 nm. For TFC, 30 μL of extract was mixed with 80 μL of 20 g L^-1^ AlCl_3_, reading absorbance at 415 nm. The TPC was expressed as mg of gallic acid (GA) kg^-1^ FW, while the TFC was expressed as mg of rutin equivalents (RE) kg^-1^ FW. Each sample extract was analyzed in triplicate.

Total antioxidant capacity (TAC) was analyzed from the same extract prepared for TPC by the 2,2-diphenyl-1-picrylhydrazyl (DPPH) method by Castillejo et al. (2021). The TAC was measured by variations in absorbance at 515 nm (Tecan Infinite M200, Mannedorf, Switzerland). TAC was expressed as mg of Trolox equivalents (TE) kg^-1^ FW. Each sample extract was analyzed in triplicate.

### 2.9 Experimental design and statistical analysis

A randomized complete block design with three replicates per nutrient solution and melatonin concentration. Each combination of nutrient solution/MEL was carried out in 135 cm x 125 cm x 20 cm beds located at three places inside a greenhouse for all the experiments. Each bed had four floating trays. A multivariate analysis of variance of agronomical and biochemical parameters (two-way ANOVA) was performed between the nutrient solution and melatonin application. When significant interactions were incorporated into the ANOVA, a least significant difference test was performed to compare how nutrient/aqueous extract and melatonin were applied. Means were compared by Tukey’s test (p ≤ 0.05) using Statgraphics Centurion (Stat Point Technologies, Inc., Warrenton, VA, USA).

## 3 Results and discussion

### 3.1 Initial chemical composition nutrient solutions and time-course of the pH and EC during *Salicornia* cultivation

The highest ionic concentration was observed in plant in T1, except for phosphorus. This deviation is likely due to the decreased availability of P with increasing pH in the nutrient solution (Cerozi da Silva and Fitzsimmons, 2016), resulting in a 90% lower concentration compared to those plants in T2 and T3. N-NH_4_^+^ was 35 and 65% lower in T1 and T2, respectively, compared to T3 (**Table 1**). Sodium and chloride concentrations of in T1 and T2 were twice as high as those in T3 and T4. Sulfate in T1 and T2 was three and fourfold higher than the T3. T1 treatment showed more saline and alkaline root environment compared to T2, which maintained an acidic root environment with moderate EC. Some experiments have been carried out in *Salicornia* spp. using different pH ranges in the NS, ranging from 5.5 to 5.8 (Moatabarniya et al., 2022; Puccinelli et al., 2024), 7.0 (Lv et al., 2011) and above 8.01 (Singh et al., 2014; Okudur and Tuzel, 2023), have shown good productive results. Particularly, Constantin et al. (2023) found no significant differences in *S. europaea* L. growth and development at different pH levels (5.29 to 8.52). As far as salinity levels are concerned, the optimal salinity for most halophyte crops ranges from 50 to 250 mM NaCl (5 to 25 dS m^-1^), showing a better growth and development in high salinity environments, with optimal values around 200 and 400 mM NaCl in some halophytes (Lombardi et al., 2022; Puccinelli et al., 2024). In our experiment, the highest salinity level used was 4.54 (dS m^-1^) in T1, close to the lowest value above, while the rest of NS had moderate and lower salinity values.

**Figure 1:**
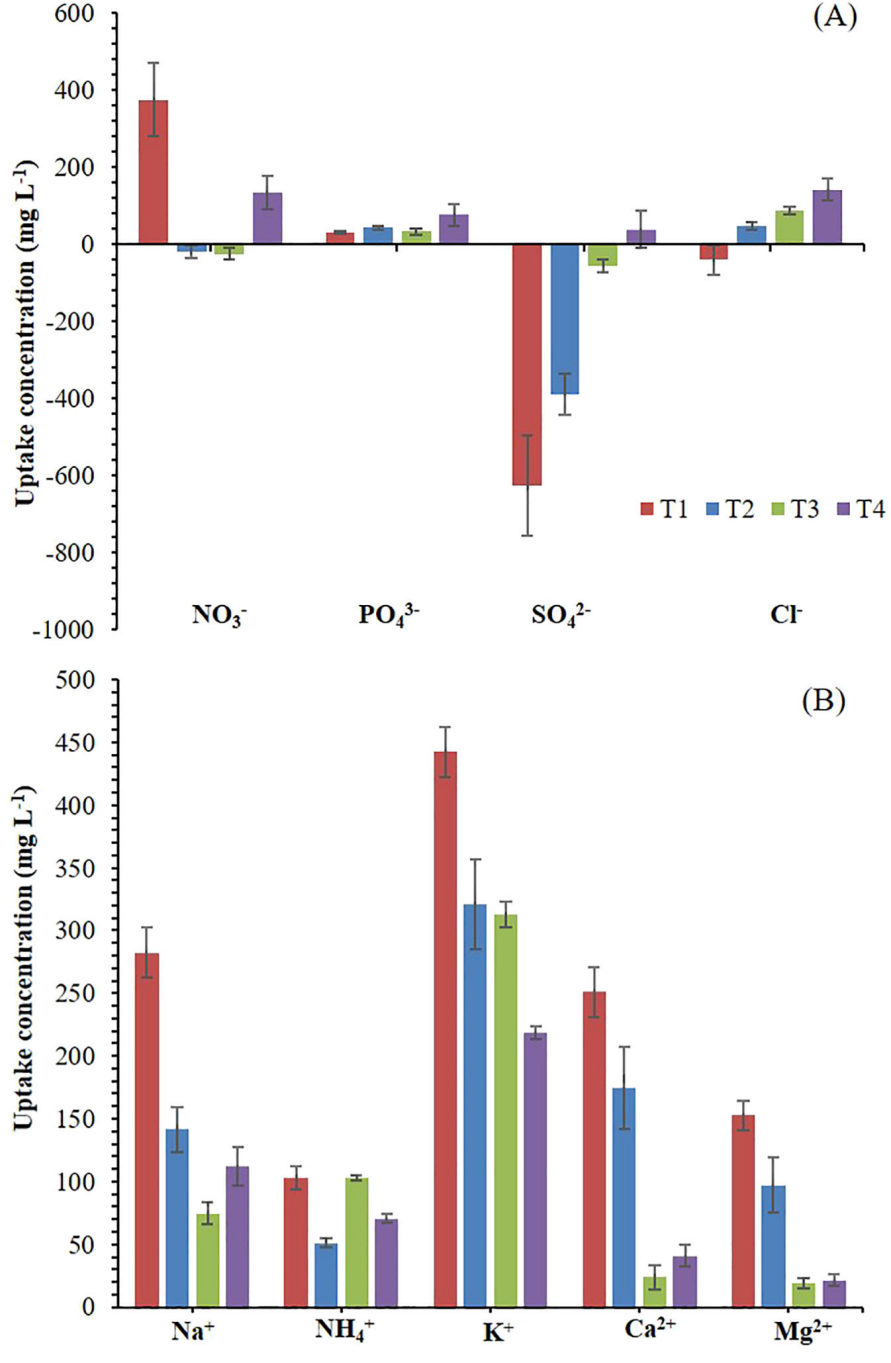
Absorption and accumulation (negative values) of anions **(A)**, and cations **(B)** at the end of the experiment. T1. compost leachate; T2. peat leachate; T3. 100% nutrient solution (NS) and T4. 50% NS strength. Vertical bars represent standard error (n=3).

Using a crop’s leachate as a nutrient solution for another crop may cause a potential nutrient imbalance and trigger precipitation and antagonism (Sambo et al., 2019; Savvas et al., 2009). Thus, T1, T2, and T3 display an inverse relationship between the absorption and accumulation of NO_3_^-^ and Cl^-^ (**Figure 1A**), likely due to the high presence of Cl^-^ in NS (**Table 2**), while in T4, this trend was probably not observed by the higher ratio Cl^-^/NO_3_^-^. Santos and Rios (2016) suggest that in irrigation water with a high Cl^-^ content, it is advisable to increase the concentration of NO_3_^-^ to mitigate the adverse effects of Cl^-^ on plants. Meanwhile, T1and T2 showed an inadequate K^+^/(Ca^2+^ + Mg^2+^) ratio, which should range between 0.5 to 1 in the NS recipe (Savvas et al., 2013; Santos and Rios, 2016), enhancing Ca^2+^ and Mg^2+^ absorption and reducing their accumulation in the solution medium (**Figure 1B**).

**Table 2:** Nutrient ratios in the nutrient solution used.

Ratios	T1	T2	T3	T4
**NH_4_^+^ N/Total-N**	0.23 ± 0.00 c	0.16 ± 0.00 d	0.4 ± 0.00 a	0.37 ± 0.00 b
**NO_3_^-^/NH_4_^+^**	3.26 ± 0.04 b	5.39 ± 0.03 a	1.5 ± 0.03 d	1.72 ± 0.01 c
**Cl^-^/NO_3_^-^**	0.92 ± 0.00 b	0.86 ± 0.00 c	0.64 ± 0.00 d	1.17 ± 0.00 a
**Ca^2+^/Mg^2+^**	0.82 ± 0.00 c	1.01 ± 0.01 b	1.18 ± 0.01 a	1.28 ± 0.13 a
**SO_4_^2-^/NO_3_^-^**	2.65 ± 0.12 a	2.34 ± 0.02 b	0.94 ± 0.00 c	1.01 ± 0.00 c
**Na^+^/K^+^**	0.61 ± 0.01b	1.26 ± 0.07 a	0.58 ± 0.01 b	1.18 ± 0.01a
**K^+^/Ca^2+^**	0.72 ± 0.01 c	0.44 ± 0.02 d	1.55 ± 0.01 a	0.99 ± 0.01 b
**K^+^/(Ca^2+^ + Mg^2+^)**	0.33 ± 0.00 c	0.22 ± 0.01 d	0.84 ± 0.00 a	0.56 ± 0.03 b

T1, compost leachate; T2, peat leachate; T3, 100% nutrient solution (NS) and T4, 50% NS strength. All data represent the mean value ± SE (n=3). Different letters in rows indicate significant differences among treatments at p ≤0.05 (Tukey´s test).

**Figure 2:**
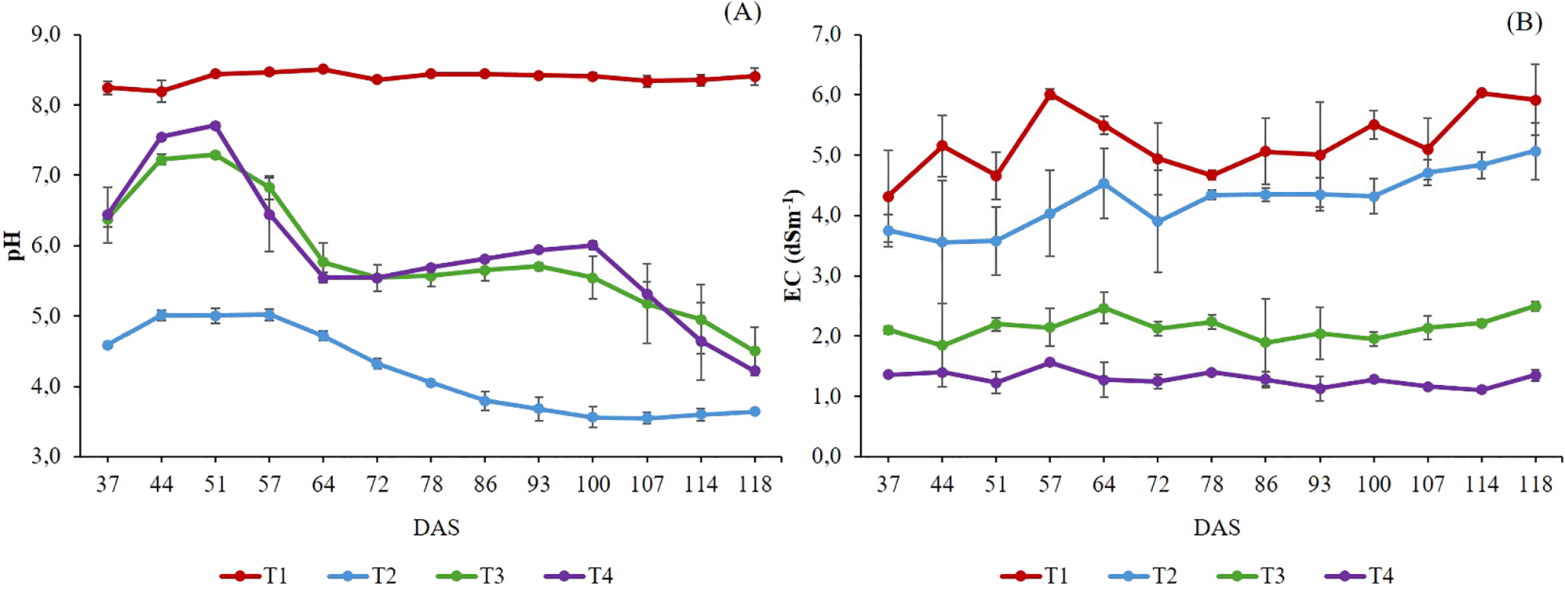
pH **(A)** and EC (dS m^-1^) **(B)** evolution in the nutrient solution and aqueous extract treatments. DAS: Days after sowing. T1. compost leachate; T2. peat leachate; T3. 100% nutrient solution (NS) and T4. 50% NS strength. Vertical bars represent standard error (n=3).

The pH value in T1 remained constant at around eight throughout the cultivation cycle. In contrast, T3, T4, and T2 showed a noticeable pH drop in the NS solution from 64 DAS onwards (**Figure 2A**), which could influence plant nutrient uptake and accumulation of ions in the NS (De Rijck and Schrevens, 1997). These pH changes could be caused by the difference in N absorption and utilization among plants since the pH value of NS varies when different NH_4_^+^/NO_3_^-^ ratios are used (Zhu et al., 2021). For instance, in T1, the most significant absorption of N-NO_3_^-^ was observed (**Figure 1A**). This likely contributed to an alkalinizing effect in the rhizosphere due to a substantial release of OH^-^ ions to maintain charge balance (Santos and Rios, 2016). In contrast, there was a higher absorption of cations in T2 which resulted in a notable reduction in pH (**Figure 2A**). The EC increased by 36% in T1 and T2 and by 20% in T3 at the end of the experiment compared to the initial values (**Figure 2B**). This was primarily due to ions’ selective absorption and accumulation (**Figure 1**), while EC in T4 remained constant throughout the crop cycle. Nevertheless, these EC values are considerably lower than those regarded as optimal for the cultivation of *Salicornia* spp., which is classified as a halophyte plant with the capability to adapt to salinity environments (Lombardi et al., 2022; Gruda et al., 2024b). In this regard, Cárdenas-Pérez et al. (2022) found that low or moderate salinity levels (2 dS m^-1^) for cultivating *Salicornia* spp. plants may decrease their productivity and biomass accumulation.

### 3.2 Water and nitrogen use efficiency

**Figure 3:**
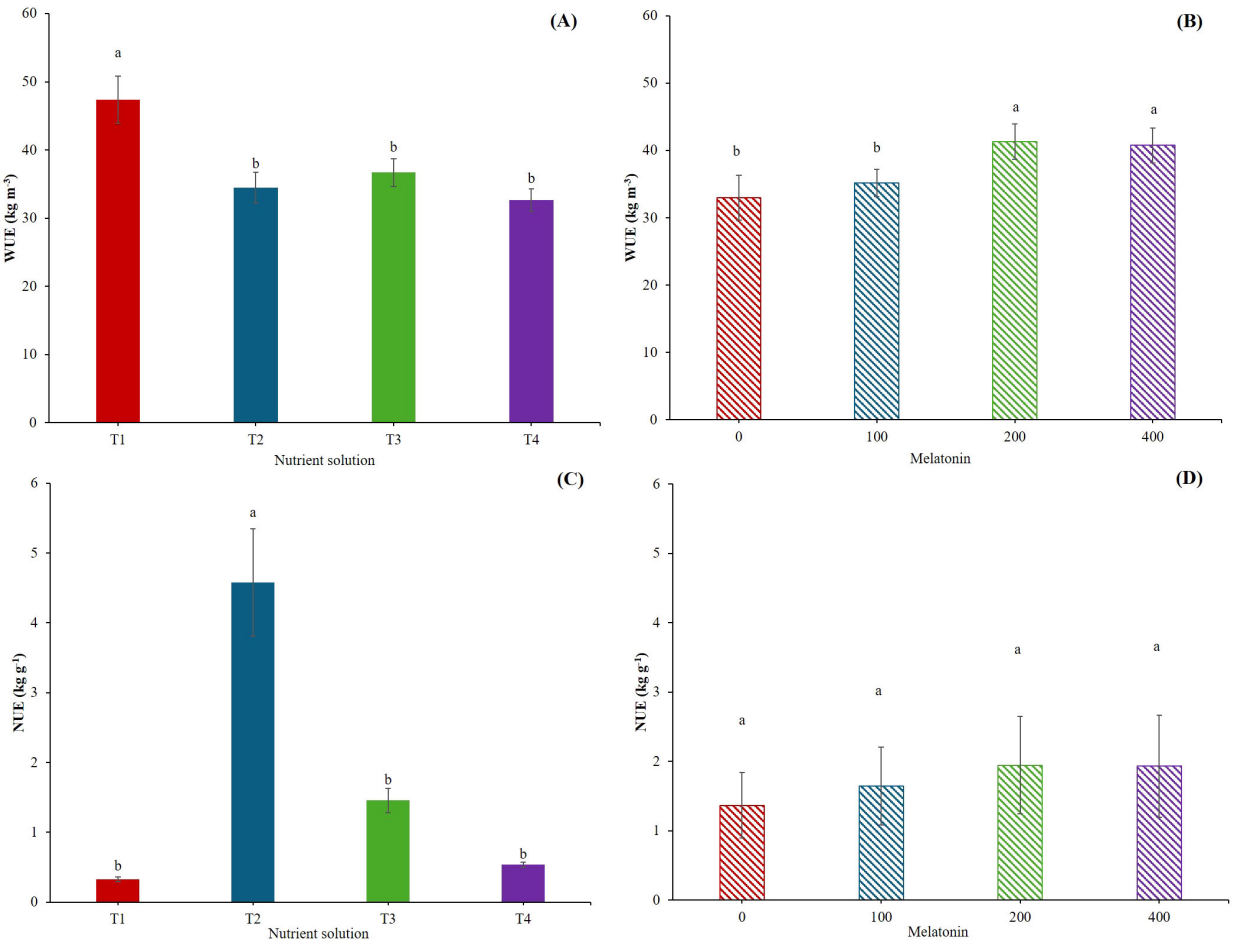
Water use efficiency (WUE, kg m^-3^) **(A, B)**, Nitrogen use efficiency (NUE, kg g^-1^) **(C, D)**. T1, Compost leachate; T2, Peat leachate; T3, 100% nutrient solution (NS) and T4, 50% NS strength. Melatonin (MEL0: 0 μM, MEL100: 100 μM, MEL200: 200 μM, MEL 400: 400 μM). Asterisk indicates significances *; ***; and n.s. at p ≤ 0.05, p ≤ 0.001 and n.s., non-significant, respectively.

The WUE parameter encompasses several meanings and relate to different approaches, such as the eco-physiological and agronomic ones, which are deeply linked (Katerji et al., 2008). In our case, we have considered the latter one, as it gives better insight into the water consumption needed to produce one kilogram of product. In our experiment, plant grown in T1 showed an average of 32% higher WUE than the other treatments (**Figure 3A**). It has been reported that mild water and salt stress may improve WUE (Liao et al., 2022), even if the salinity reduces water uptake and productivity of crops (Khataar et al., 2018). Our results are in agreement with the results reported by Kong and Zheng (2014), who observed that *Salicornia bigelovii* benefits of Na up to certain concentrations. These concentrations are similar to of Na level in T1 in our experiment, approaching to the upper limit for optimal WUE as reported by Kong and Zheng (2014). Despite T1 having the highest EC, the WUE value was not affected (**Table 1**). The reason probably lies in the adopted cultivation system, which may have played a decisive role in minimizing salinity stress and allowing the correct uptake of plant nutrients. This lack of stress at the root level and the higher uptake of nutrients by T1 probably produced a higher WUE (**Figure 3A**). In our system, the hydraulic conductivity was not limiting, as the roots were submerged in the nutrient solution. As reported by Savvas et al. (2007) and Signore et al. (2016), maintaining optimal hydraulic conductivity is of fundamental importance, as it may increase the unsaturated hydraulic conductivity and improve water availability at the root surface. 200 and 400 µM melatonin enhanced the WUE (**Figure 3B**). It has been demonstrated that the exogenous application of melatonin influences some physiological processes by regulating photosynthesis and stomatal conductance, therefore increasing water use efficiency (Arnao et al., 2022b; Jensen et al., 2023).

Unlike the WUE, the NUE was the highest in T2 (**Figure 3C**). This is not surprising since the percentage of DM in T2 was the greatest. However, other factors may influence the NUE, being the pH one of the most important parameters, with an optimum pH around 6 (Zhu et al., 2023). In our experiment, T1 and T2 were above and below, respectively, of such value, but it seems that the basic environment influenced more negatively the NUE. Apart from pH, phosphorus availability does increase NUE (Zhu et al., 2023), and the values of phosphorus in T2 were twelve-fold than that in T1 (**Table 1**), leading to a higher NUE in T2. Finally, excessive application of nitrogen leads to lower the NUE (Zhang et al., 2008). In our case, the concentration of nitrogen was significantly lower in T2 than in T1 (**Table 1**). This factor, jointly with the pH and the differences in phosphorus concentration, may explain the higher NUE in T2. There were no significant differences with melatonin application (**Figure 3D**). However, a tendency to increase NUE with the exogenous application of melatonin was observed because melatonin regulates the path of nitrogen metabolism in plants (Arnao et al., 2022c).

### 3.3 Yield and growth parameters in *Salicornia*

The highest yield was recorded in plants grown with T1 as a nutrient solution (**Table 3**). Similarly, plants in T1 had the highest root length and the S/R ratio. DM showed a significant increase when *Salicornia* was grown with peat leachate. The SSA and SAR ratios and succulence parameters were significantly increased in *Salicornia* when T3 and T4 nutrient solutions were used compared to leachates (**Table 3**). In this respect, Na^+^ may have made plants grown in T1, T3, and T4 were more succulent than those in T2 (**Table 3**). The importance of Na^+^ for the succulence of *Salicornia* plants has been highlighted by Ahmad et al. (2013), and in our case, even if the amount of Na^+^ in T1 and T2 was not different (**Table 1**), the amount of Na^+^ absorbed in T1 was almost the double than in T2 (**Figure 1B**).

**Table 3:** Influence of nutrient solution (T1, compost leachate; T2, peat leachate; T3, 100% nutrient solution (NS); T4, 50% NS strength) and melatonin doses application (MEL0: 0 µM, MEL100: 100 µM, MEL200: 200 µM, MEL400: 400 µM) on plant biometric ratios or indices (yield, DM, root length, S/R DW, specific shoot area (SSA), shoot area ratio (SAR) and succulence) of *Salicornia* cultivated in a floating system.

	Plant Biometric Ratios or Indices						
	Yield (kg m^-2^)	DM (%)	Root length (cm)	S/R DW ratio	Specific shoot area (SSA; cm^2^ g^-1^)	Shoot area ratio (SAR; cm^2^ g^-1^)	Succulence (Shoot FW/DW)
Nutrient solution (A)							
**T1**	5.41 ± 0.25 a	6.22 ± 0.07 b	17.80 ± 0.50 a	8.38 ± 0.62 a	67.52 ± 1.13	57.93 ± 1.06	16.34 ± 0.19 a
**T2**	3.67 ± 0.13 b	9.37 ± 0.57 a	14.59 ± 0.51 b	5.07 ± 0.45 b	56.20 ± 1.26	45.24 ± 1.06	11.49 ± 0.18 b
**T3**	3.91 ± 0.13 b	6.24 ± 0.12 b	15.13 ± 0.34 b	5.06 ± 0.15 b	88.96 ± 1.37	72.68 ± 1.30	16.49 ± 0.23 a
**T4**	3.48 ± 0.14 b	6.74 ± 0.14 b	14.94 ± 0.33 b	4.96 ± 0.28 b	89.82 ± 3.48	70.93 ± 2.54	16.03 ± 0.62 a
Melatonin (B)							
**MEL0**	3.51 ± 0.18 b	7.06 ± 0.15	14.72 ± 0.45	5.86 ± 0.38	77.83 ± 2.04	63.03 ± 1.47	14.87 ± 0.29
**MEL100**	4.22 ± 0.22 a	6.92 ± 0.16	15.74 ± 0.41	5.59 ± 0.53	76.78 ± 2.62	62.25 ± 1.96	15.41 ± 0.44
**MEL200**	4.40 ± 0.16 a	7.03 ± 0.15	15.71 ± 0.48	5.87 ± 0.28	74.77 ± 2.84	61.65 ± 2.29	15.25 ± 0.53
**MEL400**	4.34 ± 0.16 a	7.57 ± 0.59	16.23 ± 0.41	6.15 ± 0.49	73.13 ± 2.12	59.85 ± 1.79	14.82 ± 0.31
Significance							
**A**	***	***	***	***	***	***	***
**B**	***	n.s.	n.s.	n.s.	n.s.	n.s.	n.s.
**AxB**	n.s.	n.s.	n.s.	n.s.	**	***	n.s.

Asterisk indicates significances at **p < 0.01; ***p < 0.001; n.s, non-significant. Different letters indicate significant differences. Values are the mean ± SE (n = 3).

The application of melatonin significantly increased the yield (**Table 3**), while it did not affect the rest of the measured parameters, with a rating of 6.08 kg m^-2^ (T1/MEL100) to 2.73 kg m^-2^ (T2/MEL0) (data not shown). Melatonin has the potential to perform various functions and exert many effects on plants (Altaf et al., 2021), improving the overall growth of plants (Hernández-Ruiz et al., 2005; Murch et al., 2001).

**Figure 4:**
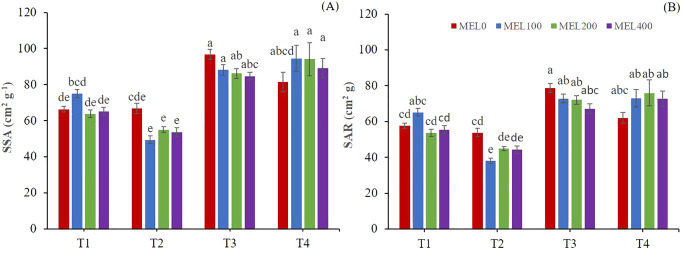
Specific Shoot ratio (SSA) **(A)** and Shoot area ratio **(B)**. T1. compost leachate; T2. peat leachate; T3. 100% nutrient solution (NS) and T4. 50% NS strength. Different letters indicate significant differences among treatments at p ≤0.05 (Tukey´s test). Vertical bars represent standard error (n= 30).

Two-way ANOVA showed a significant interaction between the nutrient solution and melatonin for SSA and SAR (**Table 3**). T1 and T2 nutrient solutions showed significantly lower SSA and SAR than T3 and T4 nutrient solutions (**Figure 4**). A lower SSA is indicative of thicker shoots, which could mean a more remarkable ability to resist postharvest stress, while a lower SAR is revealing a high plant efficiency to produce dry matter (Gallegos-Cedillo et al., 2021). In **Figure 4**, a MEL-promoting response is observed in T4 (50% NS strength) with optimum at 100-200 µM. When low NO_3_^-^ and SO_4_^2-^ were disposable, a MEL-promoting uptake was activated as has been described in other cases (Arnao et al., 2022c), where many examples on the role of MEL in different soil nitrogen condition were studied. Other positive data in **Figure 4** implies T1 at 100 µM MEL, where T1 solution presented excess of cations: K^+^, Ca^2+^ and Mg^2+^, which an osmotic stress MEL-mediated response could be implied.

### 3.4 Color evaluation and total chlorophyll and carotenoid contents

The shoot color of Salicornia plants was affected by the NS composition and melatonin concentration application. Plants grown in T1 exhibited the lowest hue angle values and the highest Chroma value, indicating reduced greenness but increasing saturation compared to the other treatments (**Table 4**). The application of MEL100 resulted in the highest of hue angle value, indicating a greener color in the plants.

**Table 4:** Influence of nutrient solution (T1, compost leachate; T2, peat leachate; T3, 100% nutrient solution (NS); T4, 50% NS strength) and melatonin doses application (MEL0: 0 µM, MEL100: 100 µM, MEL200: 200 µM, MEL400: 400 µM) on color indices (Hue and Croma) total chlorophyll content and total carotenoid content, of *Salicornia* cultivated in a floating system.

	Hue	Chroma	Total Chlorophyll Content (mg g^-1^ FW)	Total Carotenoid Content (mg g^-1^ FW)
Nutrient Solution (A)				
**T1**	122.23 ± 0.38 b	23.53 ± 1.06 a	0.23 ± 0.00 c	0.06 ± 0.00 b
**T2**	125.25 ± 0.65 a	21.51 ± 1.29 ab	0.34 ± 0.01 b	0.08 ± 0.00 a
**T3**	125.18 ± 0.49 a	19.57 ± 0.70 b	0.39 ± 0.01 a	0.08 ± 0.00 a
**T4**	124.96 ± 0.34 a	19.58 ± 1.03 b	0.37 ± 0.00 ab	0.08 ± 0.00 a
Melatonin (B)				
**MEL0**	124.23 ± 0.44 ab	21.09 ± 1.37	0.32 ± 0.02	0.08 ± 0.00
**MEL100**	125.65 ± 0.69 a	20.03 ± 1.34	0.32 ± 0.02	0.08 ± 0.00
**MEL200**	124.03 ± 0.55 ab	21.49 ± 0.76	0.35 ± 0.02	0.08 ± 0.00
**MEL400**	123.71 ± 0.53 b	21.58 ± 0.98	0.33 ± 0.01	0.07 ± 0.00
Significance				
**A**	***	*	***	***
**B**	*	n.s.	n.s.	n.s.
**AxB**	n.s.	n.s.	n.s.	n.s.

Asterisk indicates significances at *p < 0.05; ***p < 0.001; n.s, non-significant. Different letters indicate significant differences. Values are the mean ± SE (n = 3).

Chlorophylls and carotenoids are critical phytochemical compounds found in *Salicornia* with anti-carcinogenic and chemo-protective properties (Mitra et al., 2021; Milani et al., 2017; Hayes and Ferruzzi, 2020; Antunes et al., 2021). The chlorophyll assessment provides a measure of the green vegetable color consumers prefer. The nutrient solution but not melatonin applications affected the chlorophyll and carotenoid contents in this species, as in the color parameters (**Table 4**). The plants grown in T1 showed the lowest total chlorophyll and carotenoid contents and hue angle. In addition, these plants were the yellowest, as mentioned above, probably due to faster plant growth (Lara et al., 2011) and/or the high EC of T1 (**Table 1**), which could affect photosynthetic pigments (Cárdenas-Pérez et al., 2022). These data revealed a positive correlation between the hue angle and the chlorophyll content (R2 = 0.89; p < 0.01), as was expected (Neilson et al., 2013).

### 3.5 Membrane damage

**Figure 5:**
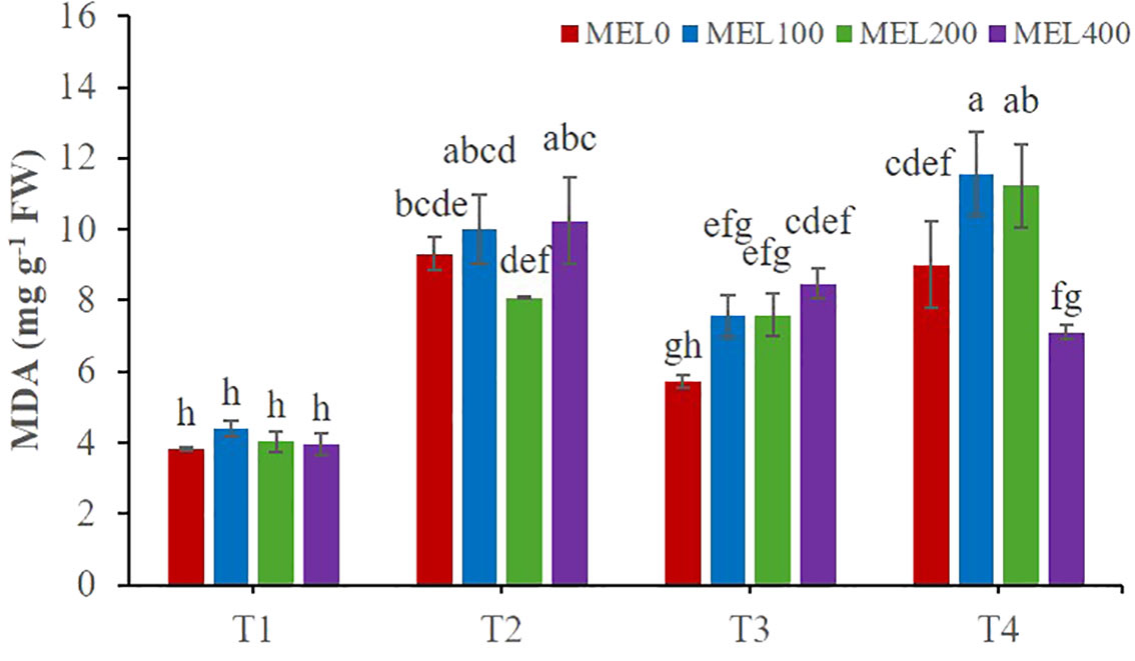
Malondialdehyde (MDA) in shoots of *Salicornia fruticosa* treated with nutrient solution (T1. compost leachate; T2. peat leachate; T3. nutrient solution (NS); T4. 50% NS strength). Different letters indicate significant differences. Vertical bars represent standard error (n=3).

Malondialdehyde (MDA), a final product of lipid peroxidation and a marker of oxidative stress in plants, was determined. *Salicornia* shoots were significantly affected by the application of nutrient solution and melatonin dose concerning the MDA content (**Table 5**). Plants grown in T4 with the application of 100 and 200 µM melatonin had the highest MDA content (11.56 and 11.23 mg g^-1^ FW, respectively) (**Figure 5**). However, the results obtained for the MDA in T1 were significantly lower in every MEL concentration applied (**Figure 5**). These results confirm those of Puccinelli et al. (2024), where *Salicornia europaea* grown under saline conditions obtained lower MDA values in treatments with higher EC, indicating a homeostatic effect induced by saline media. This fact is clearly improved by the effect of MEL acting as a homeostatic regulator, a process widely demonstrated (Arnao et al., 2022b).

**Table 5:** Malondialdehyde (MDA) in shoots of *Salicornia fruticosa* treated with nutrient solution (T1, 100% compost leachate; T2, peat leachate; T3, 100% nutrient solution (NS); T4, 50% NS strength) and melatonin doses application (MEL0: 0 µM, MEL100: 100 µM, MEL200: 200 µM, MEL400: 400 µM) cultivated in a floating system.

	MDA (mg g^-1^ FW)
Nutrient Solution (A)	
**T1**	4.06 ± 0.12
**T2**	9.40 ± 0.43
**T3**	7.25 ± 0.36
**T4**	9.72 ± 0.70
Melatonin (B)	
**MEL0**	6.96 ± 0.74
**MEL100**	8.38 ± 0.88
**MEL200**	7.70 ± 0.82
**MEL400**	7.39 ± 0.74
Significance	
**A**	***
**B**	n.s.
**AxB**	**

Asterisk indicates significances at **p < 0.01; ***p < 0.001; n.s, non-significant. Different letters indicate significant differences. Values are the mean ± SE (n = 3).

### 3.6 Ion content on shoots

The accumulation of nitrates and oxalates in the shoots was higher in T1 than in plants grown in T2 and T3. In comparison, the nitrate values in T4 were 94% higher than those of T2 and T3, on average (**Table 6**). The application of melatonin did not significantly affect its accumulation.

**Table 6:** Influence of nutrient solution (T1, compost leachate; T2, peat leachate; T3, 100% nutrient solution (NS); T4, 50% NS strength) and of melatonin doses application (MEL0: 0 µM, MEL100: 100 µM, MEL200: 200 µM, MEL400: 400 µM) on ion content (NO_3_^-^, PO_4_^3-^, Cl-, SO_4_^2-^, C_2_O_4_^2-^, Na^+^, K^+^, Ca^2+^, Mg^2+^) (mg kg^-1^ FW) and Na^+^/K^+^ of Salicornia cultivated in a floating system.

	NO_3_^-^	PO_4_^3-^,	Cl-	SO_4_^2-^,	C_2_O_4_^2-^	Na^+^	K^+^	Na^+^/K^+^ ratio	Ca^2+^	Mg^2+^
Nutrient Solution (A)										
**T1**	6784,12 ± 174.87 a	1095.34 ± 47.01 c	4576.49 ± 132.55 c	731.18 ± 28.35 b	3429.70 ± 162.61 a	5313.84 ± 171.85	4628.14 ± 136.04	1.15 ± 0.02 a	48.89 ± 1.94	419.92 ± 10.22 b
**T2**	1944.95 ± 114.37 c	1849.06 ± 58.21 b	8391.46 ± 261.91 a	1719.85 ± 77.26 a	1617.85 ± 108.77 c	3122.45 ± 68.83	7366.14 ± 149.41	0.42 ± 0.00 c	261.69 ± 24.29	777.33 ± 21.84 a
**T3**	2101.57 ± 102.06 c	2241.96 ± 85.84 a	5414.75 ± 156.56 b	688.06 ± 37.88 b	2043.70 ± 65.85 b	2958.33 ± 126.71	5364.97 ± 191.35	0.55 ± 0.01 b	68.81 ± 3.46	384.16 ± 13.77 b
**T4**	3918.86 ± 154.84 b	2190.69 ± 34.64 a	5113.38 ± 144.67 bc	654.57 ± 32.02 b	843.00 ± 44.84 d	1925.91 ± 62.08	6631.13 ± 202.08	0.29 ± 0.01 d	134.75 ± 5.83	321.85 ± 13.65 c
Melatonin (B)										
**MEL0**	3484.56 ± 572.44	1875.11 ± 124.96 ab	5906.42 ± 598.32	976.54 ± 163.64	1726.30 ± 252.22 b	3226.89 ± 326.45	5937.81 ± 347.01	0.59 ± 0.10	166.66 ± 38.60	481.78 ± 58.77
**MEL100**	3856.32 ± 572.21	1724.45 ± 148.93 b	5883.39 ± 434.06	1027.74 ± 156.25	2066.96 ± 322.77 ab	3333.33 ± 389.13	6029.71 ± 376.09	0.60 ± 0.09	112.95 ± 18.12	464.63 ± 60.77
**MEL200**	3763.88 ± 603.01	1831.11 ± 175.64 ab	5955.22 ± 480.66	923.31 ± 109.70	2088.96 ± 321.53 a	3380.65 ± 400.24	5960.41 ± 372.24	0.60 ± 0.10	133.22 ± 29.28	475.54 ± 56.69
**MEL400**	3644.85 ± 658.46	1939.31 ± 140.01 a	5751.05 ± 391.21	865.88 ± 130.56	2052.07 ± 293.41 ab	3390.66 ± 433.04	6054.84 ± 361.51	0.61 ± 0.09	101.32 ± 16.02	481.91 ± 45.27
Significance										
**A**	***	***	***	***	***	***	***	***	***	***
**B**	n.s.	*	n.s.	n.s.	*	n.s.	n.s.	n.s.	***	n.s.
**AxB**	n.s.	n.s.	n.s.	n.s.	n.s.	*	*	n.s.	***	n.s.

Asterisk indicates significances at *p < 0.05; ***p < 0.001; n.s, non-significant. Different letters indicate significant differences. Values are the mean ± SE (n = 3).

Considering the nitrate levels (European Commission, 2011), it is possible to appreciate that the nitrate levels of T1 and T4 are high and, in some cases, surpass the limits of lettuce. Specifically, T1 had a concentration of nitrates close to that reported to be the highest vegetable accumulator of nitrates, the rocket crop (Santamaria, 2006). However, for halophytes, there are no established reference values (Lombardi et al., 2022). Similar values of shoot NO_3_^-^ content (on average 3400 and 4000 mg kg^-1^ FW) in *Salicornia europaea* L. plants grown in pots fertigated with standard nutrient solution (2.7 dS m^-1^) have been reported by Puccinelli et al. (2024).

Shoot PO_4_^3-^ accumulation was significantly higher in T3 and T4. This increase in accumulation was probably modulated by the pH of the rhizosphere (**Figure 2**). Several authors mention that pH has important effects on the bioavailability of P for plants, increasing its availability when the pH of the rhizosphere is slightly acidic, whilst at extreme pHs (4-9) both above and below it they reduce its availability (De Rijck and Schrevens, 1997; Santos and Rios, 2016). Puccinelli et al. (2024) also observed the highest concentrations of P in shoots of *Salicornia europaea* irrigated with a standard nutrient solution (NS) with EC and pH (2.71 and 5.5-6.0, respectively), which closely align with the parameters employed in our T3 treatment, with respect to other NS used. MEL200 and MEL400 concentrations resulted in a notable increase in the accumulation of P in plant tissues (**Table 6**), whereas no significant differences were observed in comparison to the treatment control (MEL0). Arnao et al. (2022) mentioned that exogenous melatonin applications upregulate and optimize P absorption in several vegetable crops, as melatonin plays an important role in balancing ionic homeostasis under various stress conditions and influences plant mineral nutrition pathways. Oxalates are considered anti-nutritional compounds, because they can bind some minerals to from insoluble salts, rendering these minerals unavailable intestinal absorption (Santamaria et al., 1999; Norman et al., 2013). It is well known that some cultivated vegetables, namely those belonging to the *Amaranthaceae* family (including *Salicornia* spp.) may accumulate high concentrations of oxalate. In our experiment, T1 treatment had the highest oxalates (**Table 6**). However, the oxalates in T1 were lower than those reported in other vegetables, such as beetroot (Lisiewska et al., 2011) and spinach (Santamaria et al., 1999). This result is not surprising, as oxalate accumulation is linked to nitrate uptake, while ammonium decreases the formation of oxalate content by inhibiting nitrate uptake (Liu et al., 2015).

Shoot Na^+^ accumulation was significantly higher in T1. This could trigger a reduction in Ca^2+^ and Mg^2+^ accumulation in shoots, as shown in **Table 6**. Ahanger and Agarwal (2017) stated that salinity and a high presence of Na+ in the nutrient solution (**Table 1**) have influenced the metabolism, absorption, and translocation of Ca^2+^ and Mg^2+^.

T2 was statistically higher in the accumulation of Cl^-^ compared to the rest of the treatments. Its assimilation was probably conditioned by the low accumulation of nitrates (**Table 7**), since the accumulation of Cl^-^ and NO_3_^-^ show an inversely proportional relationship in all treatments. Yousif et al. (2010) mention an antagonistic effect between Cl^-^ and NO_3_^-^ in halophyte and non-halophyte plants and an inverse relationship between Na^+^ and cation uptake (K^+^, Ca^2+^, and Mg^2+^). Nevertheless, Ventura et al. (2011) observed no significant correlation between ion accumulations in *Salicornia* plants cultivated with seawater. These researchers suggest that plants possess a well-defined nutrient uptake system, which enables the compartmentalization of NaCl and maintains an efficient balance between the additional macronutrients required for growth and development.

**Table 7:** Influence of nutrient solution (T1, compost leachate; T2, peat leachate; T3, 100% nutrient solution (NS); T4, 50% NS strength) and melatonin doses application (MEL0: 0 µM, MEL100:100 µM, MEL200: 200 µM, MEL400: 400 µM) on the quality (total phenolic, total flavonoids and antioxidant capacity) of Salicornia cultivated in a floating system.

	Total Phenolic (mg GA kg^-1^ FW)	Total Flavonoid (mg Rutin kg^-1^ FW)	Antioxidant capacity (mg TE kg^-1^ FW)
Nutrient Solution (A)			
**T1**	3092.32 ± 142.59	489.96 ± 12.36 c	1699.31 ± 68.29 b
**T2**	5456.94 ± 233.99	749.79 ± 18.67 a	2771.51 ± 101.70 a
**T3**	2428.07 ± 64.18	623.60 ± 13.47 b	2810.94 ± 87.75 a
**T4**	2669.41 ± 76.69	619.99 ± 12.86 b	2843.86 ± 59.17 a
Melatonin (B)			
**MEL0**	3177.95 ± 266.10	624.90 ± 32.20 ab	2421.33 ± 175.71
**MEL100**	3442.87 ± 439.28	620.04 ± 30.94 ab	2535.44 ± 168.99
**MEL200**	3701.82 ± 440.94	652.89 ± 31.69 a	2659.97 ± 127.65
**MEL400**	3324.11 ± 377.13	585.52 ± 26.66 b	2508.93 ± 178.87
Significance			
**A**	***	***	***
**B**	**	*	n.s.
**AxB**	***	n.s.	n.s.

Asterisk indicates significances at *p < 0.05; **p < 0.01; ***p < 0.001; n.s, non-significant. Different letters indicate significant differences. Values are the mean ± SE (n = 3).

**Figure 6:**
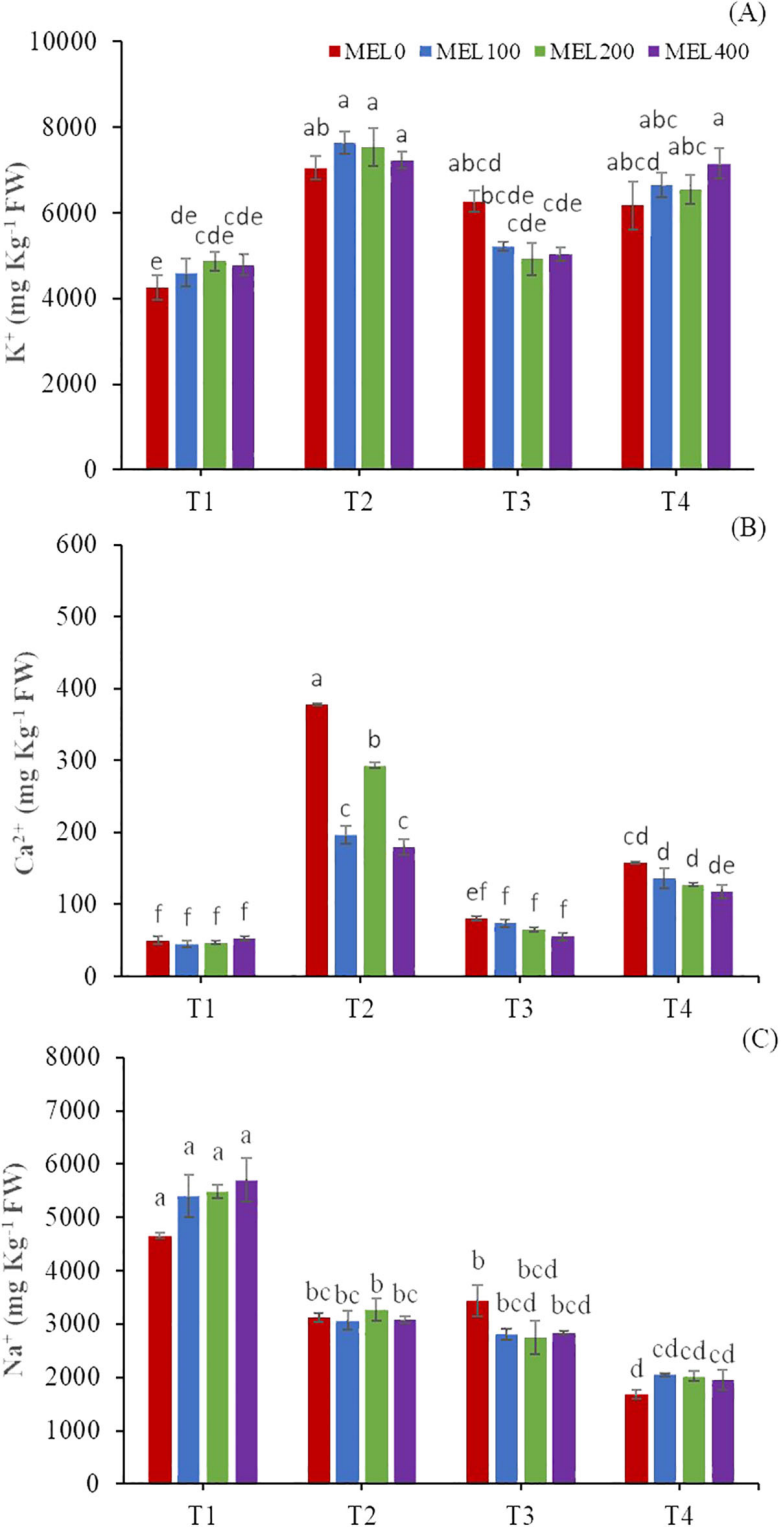
Shoots ionic content: K^+^ **(A)**, Ca^2+^ **(B)**, and Na^+^ **(C)** (mg kg^-1^ FW). T1. compost leachate; T2. peat leachate; T3. 100% nutrient solution (NS) and T4. 50% NS strength. Different letters indicate significant differences among treatments at p ≤0.05 (Tukey´s test). Vertical bars represent standard error (n= 3).

Melatonin is an essential molecule for fruit and vegetables, in particular for its role in the ripening and post-harvest processes (Xu et al., 2019), and has a role in alleviating abiotic stresses in vegetable crops (Zhou et al., 2023; Muhammad et al., 2024). In our case, the melatonin concentration only had a slight role, as it influenced the content of Na^+^, Ca^2+,^ and K^+^ jointly with the nutrient solution concentration (**Figure 6**). The application of melatonin improved the K^+^ content in all treatments except T3 (**Figure 6A**). Na^+^ content improved in T1 and T4 with the application of exogenous melatonin (**Figure 6C**) (Arnao et al., 2022b, c).

### 3.7 Total phenol content, total flavonoid content, and total antioxidant capacity

**Figure 7:**
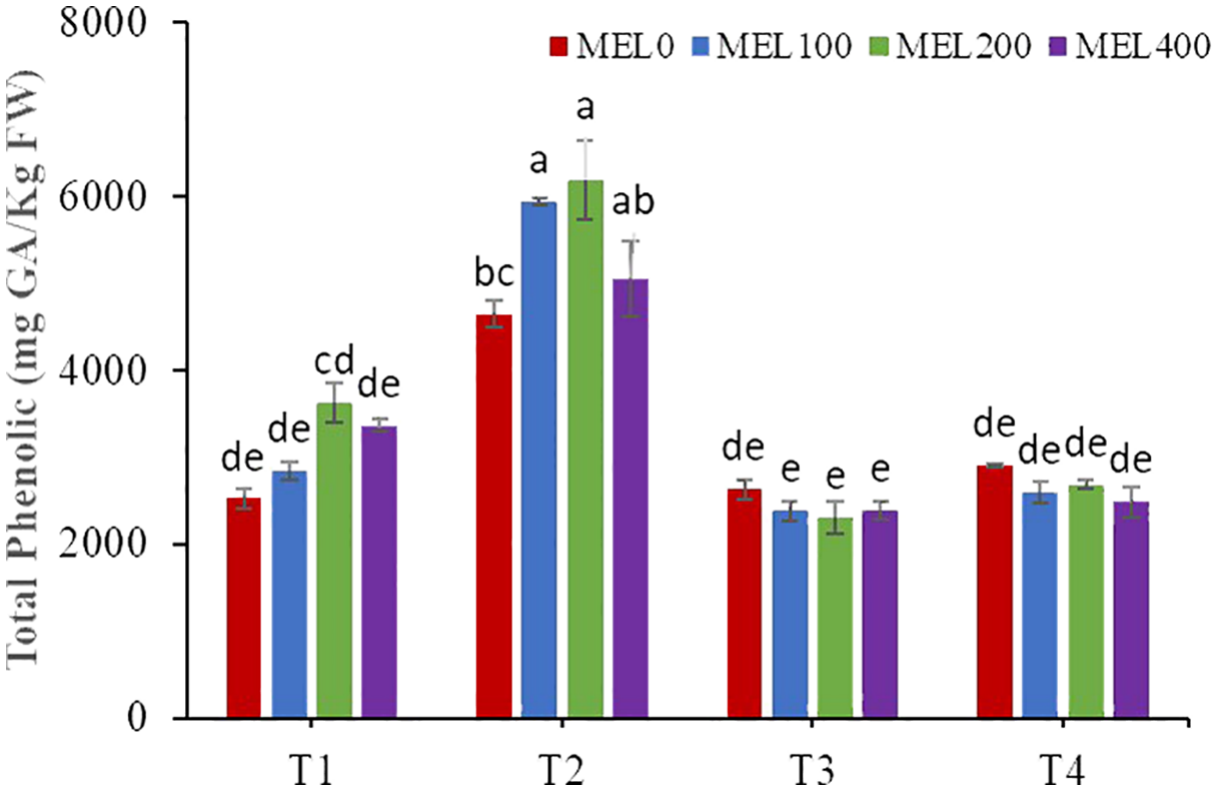
Total phenolic content in shoots of *Salicornia fruticosa* treated with nutrient solution (T1. compost leachate; T2. peat leachate; T3. 100% nutrient solution (NS); T4. 50% NS strength). Different letters indicate significant differences. Vertical bars represent standard error (n=3).

The biosynthesis of secondary metabolites in *Salicornia* may be influenced by cultivation techniques (Ventura et al., 2011). There was a significant interaction between both factors (nutrient solution composition and melatonin application) for the total phenol content (**Table 7**). Plants grown in peat leachates with 100, 200, and 400 µM melatonin had the highest total phenol content (**Figure 7**). The reduction of nutrient solution to 50% (T4) did not affect the phenol content concerning T3 independently of the melatonin application. Both factors influenced the flavonoid content, but they did not interact. The highest values were obtained in plants grown in peat leachates and in plants treated with 200 µM melatonin. Finally, the antioxidant capacity was only affected by the composition of the nutrient solution; plants grown in compost leachate showed the lowest values. In this experiment, it has been demonstrated that the cultivation techniques, such as nutrient solution composition and melatonin application, have influenced the phytochemical compound contents. Thus, the pH of the nutrient solution from peat leachate was acidic (ranging between 3 to 5), resulting in moderate oxidative stress since *Salicornia* grows in alkaline and saline soils in its natural habitat. It is well-established that plants cope with abiotic stress by altering metabolic processes, producing reactive oxygen species, and stimulating antioxidant activity to scavenge free radicals and ion chelators (Hasanuzzaman et al., 2020). Regarding melatonin treatments, 200 µM melatonin induced the highest phenolic and flavonoid contents. It is well-known that melatonin can be used to alleviate some abiotic stresses, affecting secondary metabolism (Arnao et al., 2022b) and contributing to the production of secondary metabolites more efficiently (Xu et al., 2017; Esmaeili et al., 2023). Also, it can act directly or indirectly to scavenge ROS and/or control ROS production (Arnao and Hernández-Ruiz, 2019a, b).

## 4 Conclusions

This study demonstrated that employing a CCS to collect leachates from a *Salicornia* crop and cultivate it in a floating system can enhance circularity and resource efficiency, generate ecosystem benefits, and mitigate downstream environmental contamination. The initial pH of the nutrient solution may induce moderate oxidative stress, as *Salicornia* naturally grows in alkaline and saline soils. Optimal shoot growth and performance were observed in compost leachate, although this treatment also resulted in higher concentration of nitrates and oxalates the shoots. Conversely, plants grown in peat leachates exhibited the highest total phenol content, total flavonoids, and antioxidant capacity probably as a result of the moderate oxidative stress conditions. The melatonin application presents promising potential for improving growth and shoot quality in *Salicornia* due to its role in mitigating the abiotic stresses. Despite the antinutritional aspects, *Salicornia fruticosa* offers numerous health benefits including antioxidants, phytochemicals, and other positive attributes, making it a valuable food source. These findings suggest that further research is needed to optimize the production of *Salicornia* in a floating system, focusing on reusing leachate from previous crop and incorporating melatonin to enhance cultivation practices.

## Data Availability

The raw data supporting the conclusions of this article will be made available by the authors, without undue reservation.
